# Feasibility and reliability of comprehensive three-dimensional transoesophageal echocardiography screening process for transcatheter mitral valve replacement

**DOI:** 10.1093/ehjci/jead015

**Published:** 2023-02-17

**Authors:** Francesco Piroli, Antonio Boccellino, Giacomo Ingallina, Marco Rolando, Francesco Melillo, Francesco Ancona, Stefano Stella, Federico Biondi, Anna Palmisano, Antonio Esposito, Paolo Denti, Matteo Montorfano, Francesco Maisano, Alessandro Castiglioni, Eustachio Agricola

**Affiliations:** Cardiovascular Imaging Unit, Cardio-Thoracic-Vascular Department, IRCCS San Raffaele Scientific Institute, via Olgettina 60, 20132 Milan, Italy; Cardiovascular Imaging Unit, Cardio-Thoracic-Vascular Department, IRCCS San Raffaele Scientific Institute, via Olgettina 60, 20132 Milan, Italy; Cardiovascular Imaging Unit, Cardio-Thoracic-Vascular Department, IRCCS San Raffaele Scientific Institute, via Olgettina 60, 20132 Milan, Italy; Cardiovascular Imaging Unit, Cardio-Thoracic-Vascular Department, IRCCS San Raffaele Scientific Institute, via Olgettina 60, 20132 Milan, Italy; Cardiovascular Imaging Unit, Cardio-Thoracic-Vascular Department, IRCCS San Raffaele Scientific Institute, via Olgettina 60, 20132 Milan, Italy; Cardiovascular Imaging Unit, Cardio-Thoracic-Vascular Department, IRCCS San Raffaele Scientific Institute, via Olgettina 60, 20132 Milan, Italy; Cardiovascular Imaging Unit, Cardio-Thoracic-Vascular Department, IRCCS San Raffaele Scientific Institute, via Olgettina 60, 20132 Milan, Italy; Cardiovascular Imaging Unit, Cardio-Thoracic-Vascular Department, IRCCS San Raffaele Scientific Institute, via Olgettina 60, 20132 Milan, Italy; Cardiovascular Imaging Unit, Cardio-Thoracic-Vascular Department, IRCCS San Raffaele Scientific Institute, via Olgettina 60, 20132 Milan, Italy; Cardiovascular Imaging Unit, Cardio-Thoracic-Vascular Department, IRCCS San Raffaele Scientific Institute, via Olgettina 60, 20132 Milan, Italy; Cardiovascular Imaging Unit, Cardio-Thoracic-Vascular Department, IRCCS San Raffaele Scientific Institute, via Olgettina 60, 20132 Milan, Italy; Cardiovascular Imaging Unit, Cardio-Thoracic-Vascular Department, IRCCS San Raffaele Scientific Institute, via Olgettina 60, 20132 Milan, Italy; Cardiovascular Imaging Unit, Cardio-Thoracic-Vascular Department, IRCCS San Raffaele Scientific Institute, via Olgettina 60, 20132 Milan, Italy; Cardiovascular Imaging Unit, Cardio-Thoracic-Vascular Department, IRCCS San Raffaele Scientific Institute, via Olgettina 60, 20132 Milan, Italy; Cardiovascular Imaging Unit, Cardio-Thoracic-Vascular Department, IRCCS San Raffaele Scientific Institute, via Olgettina 60, 20132 Milan, Italy

**Keywords:** TMVR, neo-LVOT, transoesophageal echocardiography

## Abstract

**Aims:**

The procedural planning of transcatheter mitral valve replacement (TMVR) requires a specific imaging assessment to establish patient eligibility. Computed tomography (CT) is considered the reference method. In this setting, data regarding the role of transoesophageal echocardiography (TOE) are lacking. We evaluated the feasibility and reliability of a comprehensive 3D-TOE screening in TMVR candidates.

**Methods and results:**

We performed a retrospective observational study including 72 consecutive patients who underwent a pre-procedural CT and 3D-TOE for TMVR evaluation. The measurements of mitral annulus (MA), length of anterior mitral leaflet (AML), native left ventricular outflow tract (LVOT), and predicted neo-LVOT acquired with CT and 3D-TOE were compared using a novel semi-automated software for post processing analysis (3 mensio Structural Heart 10.1—3mSH, Pie Medical Imaging, Bilthoven, Netherlands). The final suitability decision was given by the valve manufacturer based on CT measurements and clinical conditions. Among 72 patients screened, all patients had adequate image quality for 3D-TOE analysis. 3D-TOE and CT measurements for AML length (*r* = 0.97), MA area (*r* = 0.90), perimeter (*r* = 0.68), anteroposterior (*r* = 0.88), and posteromedial-anterolateral (*r* = 0.74) diameters were found highly correlated, as well as for native LVOT (*r* = 0.86) and predicted neo-LVOT areas (*r* = 0.96) (all *P*-values <0.0001). An almost perfect agreement between CT and 3DTOE was found in assessing the eligibility for TMVR implantation (Cohen kappa 0.83, *P* < 0.001).

**Conclusion:**

3D-TOE appraisements showed good correlations with CT measurements and high accuracy to predict TMVR screening success.

## Graphical Abstract

**Graphical Abstract:**
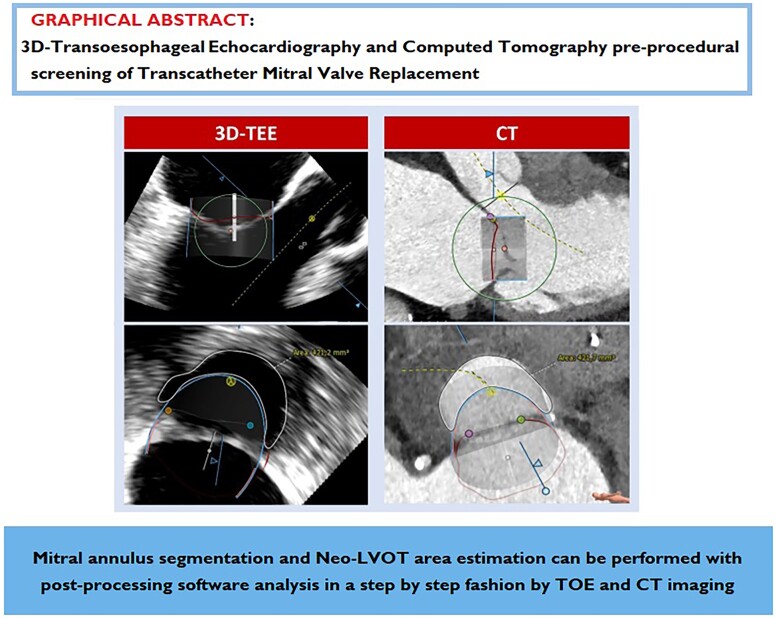

## Introduction

Transcatheter mitral valve replacement (TMVR) is emerging as a less invasive treatment than conventional surgery for high-risk patients that could overcome some limitations of transcatheter edge-to-edge repair. However, TMVR faces several challenges, including the careful selection of the device size to match the individual dimensions and geometry of the mitral annulus (MA) as well as the risk of left ventricular outflow tract obstruction (LVOTO).^1–3^ Therefore, pre-procedural planning to evaluate the mitral valve (MV) apparatus and its relationship with adjacent structures is required.^4^ Currently, echocardiography and computed tomography (CT) are the key modalities for the pre-procedural planning of TMVR. A comprehensive echocardiographic evaluation is essential to establish the mechanism and severity of mitral regurgitation (MR) and the myocardial function, while CT is crucial for the assessment of the morphology of MV apparatus and the risk of LVOTO post-TMVR.^5^ Dedicated CT software, simulating the virtual prosthesis implantation in a post-processing image analysis, demonstrated a high capability in predicting neo-LVOT areas^6^ and consequently estimating the risk of TMVR-induced LVOTO, identifying an unsuitable anatomy for the procedure. Recent studies showed that three-dimensional transoesophageal echocardiographic imaging is reliable to measure MA dimensions with good agreement compared to CT in patients referred for TMVR^7^ and demonstrated a good correlation between CT and echocardiography in LVOT area assessment.^8^ However, echocardiography has never been fully evaluated as a complementary or alternative method for the correct planning of TMVR, playing only a marginal role in the pre-procedural risk stratification. The idea of a comprehensive echocardiographic screening for the feasibility of TMVR is appealing, since it would have the potential to rapidly and safely rule out patients not suitable for the procedure, avoiding unnecessary, and potentially harmful CT evaluation.

The aim of this study was to test both the feasibility and reliability of a comprehensive echocardiographic screening in candidates for TMVR, using a novel semi-automated software platform based on 3D-transoesophageal echocardiography (TOE) (3mensio Structural Heart 10.1—3mSH, Pie Medical Imaging, Bilthoven, Netherlands) compared with CT as a reference.

## Methods

### Patient selection and study design

From 2018 April to 2021 December, we retrospectively included in the study all the consecutive patients affected by severe MR at high risk for conventional surgery with inadequate mitral anatomy for Transcatheter Edge-to-Edge Repair evaluated for TMVR in our heart valve centre (San Raffaele University Hospital) through a comprehensive CT and TOE. The exclusion criterion was poor imaging quality. The ethic committee approved the protocol (CE: 232/2016, CE: 231/2016, CE: 175/2018) for the screening procedure and all patients provided a written informed consent.

### Images acquisition

Cardiac CT imaging was performed using a second-generation dual-source scanner (Somatom Definition Flash, Siemens Healthineers, Erlangen, Germany) with a retrospective ECG-gated acquisition, during the injection of iodinated contrast media (Visipaque 320, General Electric Healthcare, Chicago, IL, USA). Multiphase images were reconstructed in a standard fashion at every 10% of the R-R interval (0–90%), at a slice thickness of 0.6 mm with an increment of 0.5 mm, using smooth kernel (I36) and an iterative reconstruction algorithm (SAFIRE, strength 2, Siemens Healthineers).

A comprehensive transthoracic and TOE exam was performed using the commercially echocardiographic ultrasound systems (Philips Epiq 7 with × 8-2t TOE probe, Philips Ultrasound Inc, Bothell, Washington, USA; GE Vivid E95 with 6VT-D TOE probe, GE Healthcare, Chicago, IL, USA) according to current guidelines.^9,10^ 3D images of mitral valves and aortic roots with a focus on LVOT were obtained using 3D zoom modalities acquired over one cardiac cycle with a frame rate between 12 and 32 volumes per second. Images were digitally stored and transferred to a workstation for offline analysis. Finally, a post-procedural transthoracic echocardiogram (TTE) was performed to assess the correct deployment of the valve and the presence of post-TMVR LVOTO. LVOTO was defined as an increase of >10 mmHg in the LVOT peak gradient by echocardiography, according to MVARC criteria.^11^

### Post-processing and measurements

The post-processing analysis of MA differed between native valve or valve in mitral annular calcification (ViMAC) and valve-in-ring (ViR) or valve-in-valve (ViV). In the setting of native TMVR or ViMAC, we performed a three-dimensional segmentation of mitral annulus on an end-diastolic frame; on the other hand, in ViV and ViR procedures, we assumed the mitral annulus as circular, therefore annular area, perimeter and diameters were derived by manufacturer's specifications.

CT data analysis was performed with a dedicated software (3mCT; 3mensio, Pie Medical Imaging, Bilthoven, Netherlands), following a step-by-step protocol suggested and already described by expert-based recommendations in a previous study as the method of choice to segment and measure MA and the predicted neo-LVOT.^12^ Briefly, the saddle-shaped MA was segmented by placing 16 seeding points along the contour of the fibrous aorto-mitral continuity and along the insertion of the posterior mitral leaflet, while the long axis view was rotated in an automated fashion every 22.5°. The segmented saddle-shaped annulus was then automatically truncated along a virtual line connecting both trigones, to obtain a D-shaped annulus.^13^ Finally, the projected area, projected perimeter, anteroposterior (AP), posteromedial-anterolateral (PM-AL) diameters, and length of anterior mitral leaflet (AML) were measured.

**Figure 1:**
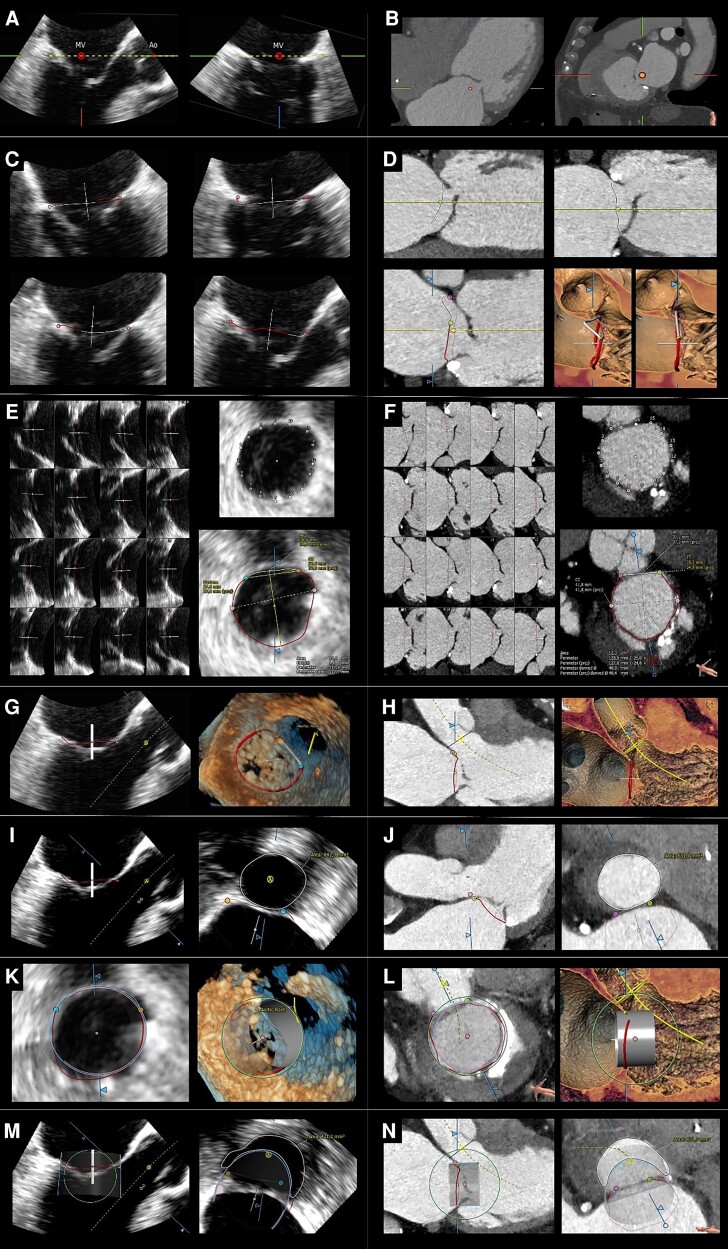
Mitral annulus segmentation and neo-LVOT area measurement performed by means of the 3mensio software (i.e. medical imaging, bilthoven, Netherlands) in a step-by-step fashion by TOE and CT imaging. *A* and *B*: preliminary orientation and centring of the mitral annulus on an end-diastolic frame. *C*: mitral annulus is traced by placing eight points on the annular hinge points on four views (4-chamber, commissural, antero-posterior and 3-chamber views). *D*: mitral annulus is traced by placing the hinge points on the long axis view of multiple reconstructions of the mitral valve; every time a hinge point is placed the long axis will automatically make a standard rotation of 22.5°. Annulus annotation is based on 16 hinge points. The saddle-shaped annulus so traced is then automatically truncated along a virtual line connecting both trigones generating a D-shaped annulus (truncation of the anterior horn is shown in the volume-rendering images). *E* and *F*: improving of annulus tracing by adding and repositioning (if necessary) the hinge points and final visualization of the annulus with projected area, perimeter and diameters annotated. *G*–*J*: after tracing a line following the trajectory of the LVOT (*G* and *H*) the native LVOT can be manually traced by planimetry on a plane orthogonal to this centreline. *K* and *L*: virtual simulation of a cylindric device implantation. M and n: the minimal systolic neo-LVOT area is finally manually measured by planimetry on a plane orthogonal to the centreline.

A similar post-processing analysis of 3D-TOE data sets was performed using a new dedicated software (3mensio Structural Heart 10.1—3mSH, Pie Medical Imaging, Bilthoven, Netherlands). First, the annulus was segmented by placing eight points on the annular hinge points on four views (4-chamber, commissural, antero-posterior, and 3-chamber views) automatically generated by the software after a preliminary alignment and centring of the mitral valve. Once the annulus was drawn, adjunctive points were eventually added and repositioned to improve the tracing. A manual truncation of the anterior horn along a virtual line connecting both fibrous trigones was then performed to generate a D-shaped annulus. To generate the minimal predicted neo-LVOT area, this same segmentation, either by CT or TOE, was repeated in end systole simulating a virtual device implantation perpendicularly to the mitral annulus. This virtual device is a cylinder whose dimensions (height, inflow diameters, and outflow diameters) were derived and modified according to the manufacturer's specifications of the valve chosen for the intervention. After drawing a line following the trajectory of the LVOT, either by CT or TOE, the minimal neo-LVOT area was manually traced at the inner edge on a plane orthogonal to this centreline^14^ (*Figure 1*).

The post-processing analyses of CT and TOE data sets were performed by one cardiologist expert in cardiovascular imaging (AB) anonymously so that CT and TOE measurements could not be compared.

### TMVR screening

The screening process based on CT imaging classifies patients as being suitable or unsuitable for TMVR. The TMVR screening for native valve was performed on three dedicated prostheses: Tendyne (Abbott Structural Heart, Santa Clara, California), Tiara (Neovasc, Richmond, Canada), and Cardiovalve (Cardiovalve Ltd., Or Yehuda, Israel). A TMVR screening for ViV and ViR was performed on two dedicated prostheses Myval (Meril Life Sciences Pvt. Ltd., Vapi, Gujarat, India) and Sapien 3 (Edwards Lifesciences Corporation; Irvine, California). In this group, the eligibility was based on the dimensions of the bioprosthesis or the ring and the CT derived assessment of LVOTO risk. For all the patients appraised, the anatomical exclusion criterion for intervention was an estimation of neo-LVOT area < 1.7 cm^2^ since this cut-off was previously described as one of the main predictors of LVOTO after TMVR.^15^ For candidates to native valve TMVR, unsuitable MA dimensions whose cut-off were indicated by the manufacturers’ specifications were also considered as criteria of exclusion.

In addition to our CT screening, final feasibility assessment was made by the manufacturer according to CT-derived measurements of MA (area, perimeter, AP diameter, PM-AL diameter), the estimated neo-LVOT area, and the clinical condition.

### Accuracy of 3D-TOE screening

The accuracy of 3D-TOE in identifying predicted neo-LVOT < 1.7 cm^2^ compared to CT as reference standard was evaluated in the entire population. In the subgroup population of candidates for native valve TMVR, the ability of 3D-TOE to identify a too small or too large MA for the feasibility of the intervention, according to the manufacturer’s cut-off dimensions, was assessed as well.

### Statistical analysis

Continuous variables are expressed as mean ± SD and categorical variables as frequencies and percentages. Normality of distributions for continuous variables was tested with the Shapiro–Wilk test. Two paired sample *t*-test or Wilcoxon test, as appropriate, were used to assess the difference between 3D-TOE vs. CT measurements. A Pearson’s correlation coefficient (*r*) and simple regression analysis were calculated to evaluate the effect size of the association between the two methods. The strength of correlation between TOE and CT was interpreted as follows: weak ≤0.39, moderate 0.40–0.69, strong 0.70–89 and very strong >0.90. Bland–Altman analysis was used to investigate the limits of agreement between the two modalities and to visualize any possible discrepancies between the measurements and the true value (i.e. proportional bias).^16^

To determine intraobserver and interobserver reproducibility, 3D-TOE measurements of 30 randomly selected patients were repeated by the same operator and by a second blinded one, and then compared using intraclass correlation coefficient (ICC). TOE screening accuracy was finally compared to CT by McNemar’s test and Cohen’s kappa. Cohen’s kappa estimates the proportion of agreement that exceeds the occurrence due to chance and was graded as slight (κ value 0.01–0.20), fair (0.21–0.40), moderate (0.41–0.60), substantial (0.61–0.80), or almost perfect (0.81–1.00).^17^ This terminology will be used to describe the concordance in the next sections. Statistical analysis was conducted using R version 3.6.2 software (R Foundation for Statistical Computing, Vienna, Austria). A two-sided *P*-value <0.05 was considered significant.

## Results

### Characteristics of the study population

Baseline clinical characteristics of the patients enrolled (*n* = 72) are listed in *Table 1*. The mean age was 79.0 years, 55% female. Mean STS score was 9.1% ± 3.1%. All patients had adequate image quality for 3D-TOE analysis.

**Table 1:** Baseline characteristics of population

Characteristic	
Age (Q1-Q3)	79 (74–83)
Female	40 (55%)
BSA (m^2^)	1.7 ± 0.21
Diabetes mellitus	21 (29%)
CKD (eGFR < 60 mL/min)	30 (42%)
History of AF	22 (30%)
EF	49 ± 7%
NYHA class	
I	5 (7%)
II	35 (48%)
III	30 (42%)
IV	2 (3%)
Mechanism of MR	Degenerative 35 (49%)
	Functional 33 (46%)
	Rheumatic 4 (5%)
STS	9.1% ± 3.1%
Euroscore II	7.4% ± 3.2%

AF, atrial fibrillation; BSA, body surface area; CKD, chronic kidney disease; EF, ejection fraction. Continuous variables are expressed as mean ± standard deviation or medians with interquartile ranges [IQRs] when appropriate. Categorical variables as frequencies and percentages.

### Comparison of 3D-TOE and CT

MA measurements are shown in *Table 2*. 3D-TOE showed a tendency to underestimate measurements compared to CT, with a mean absolute difference ranging from −0.44 ± 1.02 cm^2^ (Projected MA area) to −3.32 ± 10.61 mm (Projected MA perimeter). The correlation between CT and 3D-TOE was very strong for annulus area (*r* = 0.90, *P* < 0.001) and AML length (*r* = 0.97, *P* < 0.001), strong for AP diameter (*r* = 0.88, *P* < 0.001) and for PM-AL diameter (*r* = 0.74 *P* < 0.001) (*Table 2* and see Supplementary material:Graphs). A moderate correlation was found with the MA perimeter (*r* = 0.68 *P* < 0.001).

**Table 2:** Comparison between TOE and CT measurements

	3DTOE imaging	CT imaging	Difference *P*-value	Bland Altman 3D-TOE—CT	*R*	*P*
Native LVOT area (cm^2^)	4.32 ± 1.01	4.31 (3.69, 5.09)	0.39	−0.20 (−1.05, 0.64)	0.86	<0.0001
Neo-LVOT area (cm^2^)	2.65 (1.42, 3.72)	2.59 (1.47, 3.79)	0.76	−0.06 (−0.65, 0.52)	0.96	<0.0001
Projected MA area (cm^2^)	10.20 ± 3.18	10.64 ± 3.13	0.47	−0.44 (−2.43, 1.55)	0.90	<0.0001
Projected MA perimeter (mm)	118.87 ± 18.42	122.19 ± 16.91	0.33	−3.32 (−24.11, 17.47)	0.68	<0.0001
AP diameter (mm)	32.28 ± 7.01	33.05 ± 7.08	0.57	−0.76 (−5.61, 4.08)	0.88	<0.0001
PM-AL diameter (mm)	38.42 ± 5.31	39.06 ± 4.69	0.50	−0.64 (−5.94, 4.66)	0.74	<0.0001
AML length (mm)	25.00 (22.00, 29.00)	25.11 ± 6.34	0.59	−0.56 (−2.73, 1.60)	0.97	<0.0001

Continuous variables are expressed as mean ± standard deviation or medians with interquartile ranges [IQRs] when appropriate.

*Table 2* lists the mean values of LVOT and predicted neo-LVOT areas. 3D-TOE confirmed a little tendency to underestimate measurements compared to CT with no significant difference between the two methods. Pearson correlation coefficient demonstrated a very strong correlation for both predicted neo-LVOT area (*r* = 0.96, *P* < 0.0001) and a strong correlation for native LVOT (*r* = 0.86, *P* < 0.0001) area measurements.

Supplementary data online *Table S1* shows the correlation between CT and 3D-TOE measurements in the subgroups of patients evaluated for ViV and ViR, native anulus without severe annular calcification and ViMAC.

### Accuracy of 3D-TOE screening compared to CT as reference method

The screening process on the entire population revealed an almost perfect agreement between CT and 3D-TOE based on MA dimensions and LVOT measurements (Cohen kappa 0.83, *P* < 0.001) with excellent sensitivity and specificity (*Table 3*).

**Table 3:** Accuracy of 3D-TOE in TMVR screening compared to CT as a reference standard

	3D-TOE screening Positive	3D-TOE screening Negative	Total
CT screening positive	37	3	40
CT screening negative	3	29	32
**Total**	40	32	72

	3D-TOE
Sensitivity (95% Cl)	0.91 (0.75, 0.98)
Specificity (95% Cl)	0.92 (0.80, 0.98)
Accuracy (95% Cl)	0.92 (0.83, 0.97)
Cohen kappa (95% Cl)	0.83(0.82- 1.03)
Positive predictive value (95% Cl)	0.91 (0.75, 0.98)
Negative predictive value (95% Cl)	0.92 (0.80, 0.98)
Mcnemar's Test *P*-Value	1

In the subgroup of 56 patients evaluated for TMVR in native annulus, the screening process confirmed an almost perfect agreement between CT and 3D-TOE based on MA dimensions and LVOT measurements (Cohen kappa 0.82, *P* < 0.001) (see Supplementary data online, *Table S2*).

**Figure 2:**
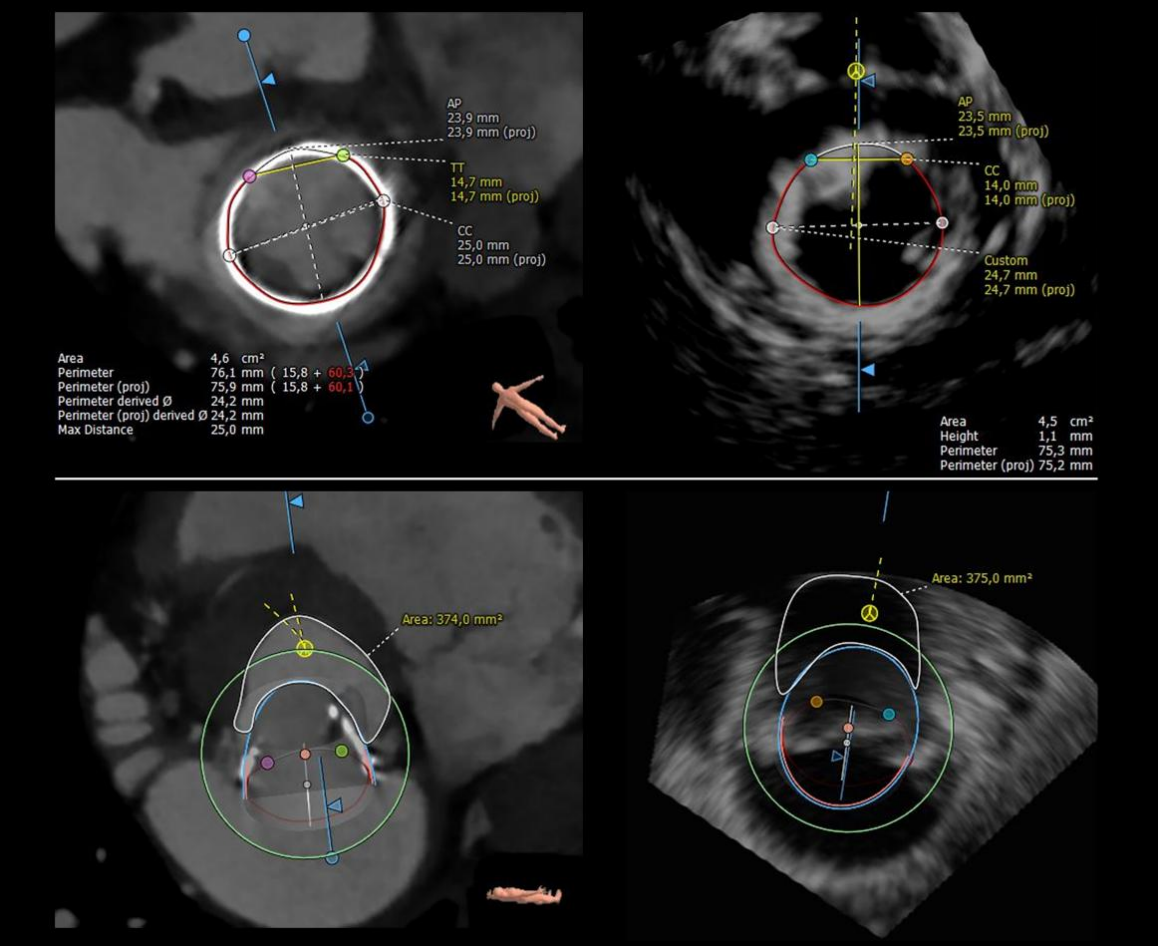
Example of a valve in valve assessment: top: segmentation of the basal ring of a 25-mm Magna bioprosthetic valve by TOE and CT imaging. Bottom: virtual simulation of a 26-mm Sapien bioprosthetic valve implantation (valve-in-valve procedure) by TOE and CT with subsequent Neo-LVOT estimation.

In the subgroup of 16 patients evaluated for VIV and VIR, the screening process showed a substantial agreement between CT and 3D-TOE based on LVOT measurements (Cohen kappa 0.76, *P* = 0.03) (see Supplementary data online*Table S3* and *Figure 2*).

**Figure 3:**
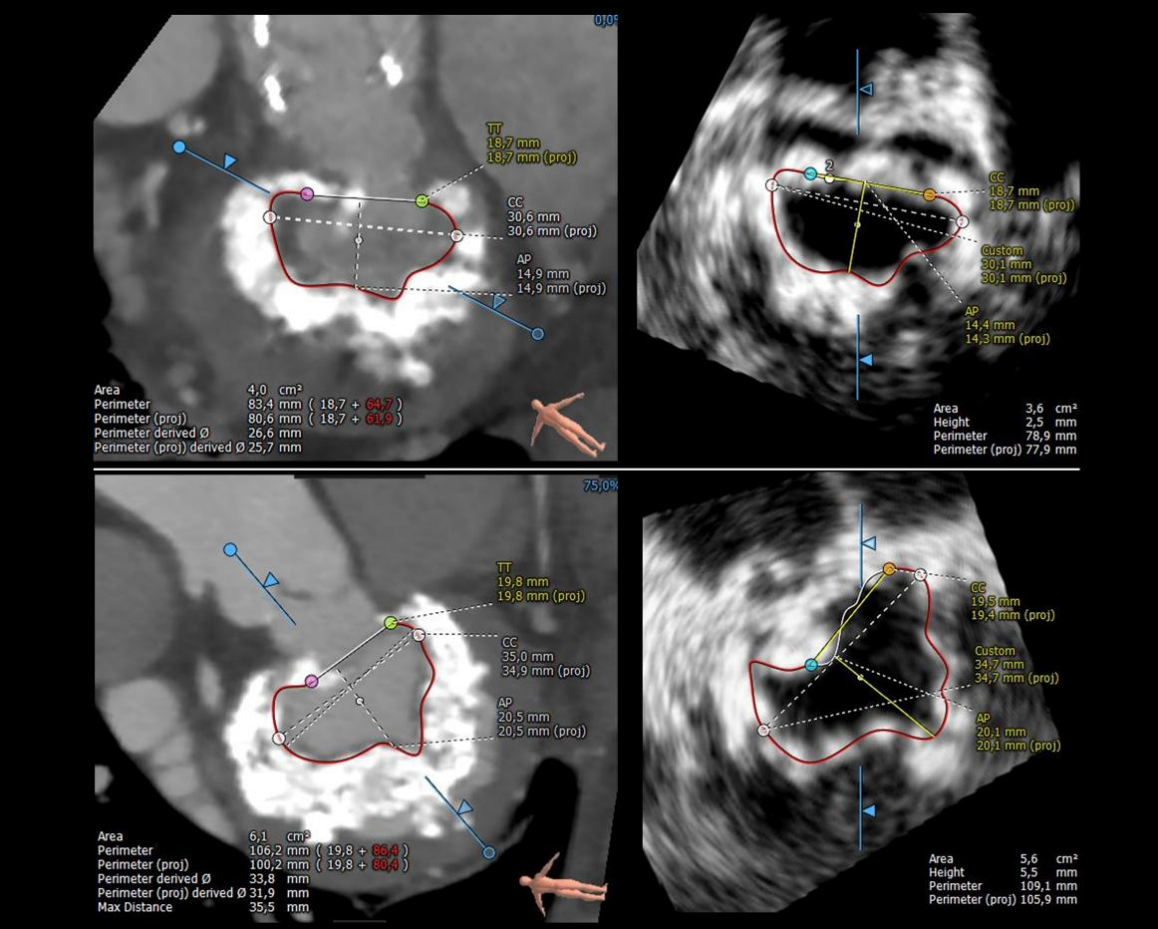
Valve in MAC: segmentation of the calcified mitral annulus by TOE and CT imaging using the D-shape method.

Finally in the subgroup of 16 patients evaluated for TMVR in MAC, where calcified mitral annulus may limit echocardiographic evaluation, the screening process showed an almost perfect agreement between CT and 3D-TOE based on MA dimensions and LVOT measurements (Cohen kappa 0.82, *P* = 0.008) (see Supplementary data online*Table S4* and *Figure 3*).

Analyzing the individual parameters separately, in the assessment of the predicted neo-LVOT area <1.7 cm^2^, the concordance of CT and 3D-TOE on the entire population was nearly perfect (Cohen kappa 0.94, *P* < 0.001) (see Supplementary data online, *Table S5*). Within the subgroups, the concordance of CT and 3D-TOE, in the assessment of the predicted neo-LVOT area <1.7 cm^2^, was perfect in ViMAC (Cohen kappa 1, *P* < 0.001), nearly perfect in TMVR in native annulus without MAC (Cohen kappa 0.94, *P* < 0.001) and substantial in ViV and ViR (Cohen kappa 0.76, *P* = 0.03).

In the subgroup of 56 patients evaluated for native TMVR, the concordance between CT and 3D-TOE was moderate in identifying the MA with too small diameter (Cohen kappa 0.56, *P* = 0.004) and excellent in identifying the MA with too large diameter (Cohen kappa 1, *P* < 0.001) (see Supplementary data online*Tables S6* and *S7*). In particular, in the 16 ViMAC patients, the concordance between CT and 3D-TOE was perfect in identifying the MA with too large diameter (Cohen kappa 1, *P* = 0.07) and substantial for the MA with too small diameter (Cohen kappa 0.71, *P* = 0.004); in the 40 TMVR patients without MAC, the concordance was perfect in identifying the MA with too large diameter (Cohen kappa 1, *P* = 0.076) and fair for the MA with too small diameter (Cohen kappa 0.26, *P* = 0.26).

### Eligibility for TMVR and procedural results

**Figure 4:**
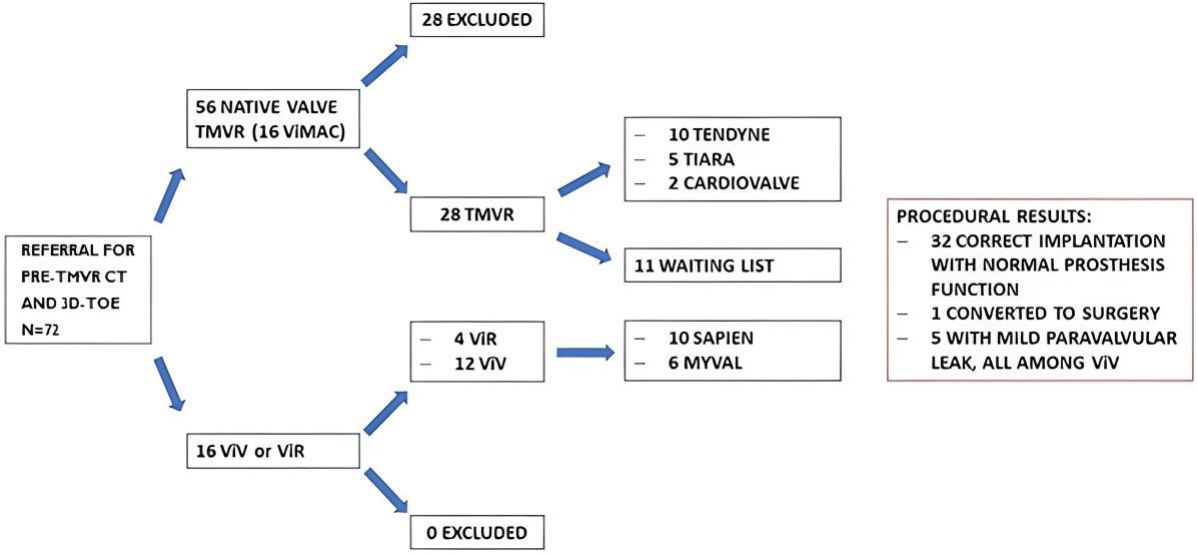
Flowchart of patients’ periprocedural management.

Of the 72 patients screened, 56 were evaluated for native valve TMVR and 16 for ViV or ViR procedures. According to the manufacturer evaluation based on CT imaging analysis and clinical condition, 28 patients were considered ineligible. Among the remaining 44 patients with a successful screening, 34 underwent TMVR and 10 are on the waiting list for the procedure. Procedures consisted in 12 ViV, 4 ViR and 18 implantations on native valve: 33 underwent a correct implantation without complications and one was converted to open-heart surgery due to the atrial migration of the prosthesis (*Figure 4*).

At post-procedural TTE evaluation, all the patients presented normal prosthesis function and a mild paravalvular leak was found in five patients. All the paravalvular leaks were detected in ViV implantations. In two patients with ViV implantation, post-procedural LVOTO was found.

Four, two and three patients underwent TMVR despite a neo-LVOT slightly smaller than 1.7 cm^2^, MA slightly smaller and slightly larger than required, respectively. Moreover, two and three patients were excluded for comorbidities and for low fossa ovalis, respectively. This explains the discrepancy between our retrospective screening results (40 suitable patients) and the final eligibility assessment by manufacturers (44 suitable patients) (see Supplementary data online*Table S8*).

One of the two patients with post-procedural LVOTO presented a predicted neo-LVOT slightly smaller than 1.7 cm^2^.

### Reproducibility

Interobserver and intraobserver agreements were found excellent for all parameters appraised with ICC >0.85 (see Supplementary data online*Table S9*).

## Discussion

The results of the present study show: (1) a comprehensive echocardiographic screening in candidates for TMVR is feasible and reliable; (2) an overall strong correlation and reproducibility for most parameters of MA, LVOT and predicted neo-LVOT areas compared to CT were found. This strong correlation is maintained in the whole population and in the subgroups of candidate patients for ViV and ViR, ViMAC and native annulus; (3) an almost perfect agreement of 3D-TOE to predict TMVR screening success as defined by manufacturer recommendations according to CT measurements.

A specific imaging evaluation focused on the MV anatomy and the relationship between MV and other nearby structures during pre-procedural TMVR screening is mandatory. Traditionally, pre-procedural 2D and 3D-TOE is performed for MR quantification, to qualitatively assess the MV apparatus and to define the mechanism of MR, but its role in pre-procedural screening has only been marginally evaluated. To date, indeed, only two studies showed the utility of 3D-TOE in TMVR sizing for a dedicated prosthesis, but they did not assess its diagnostic role in the appraisement of the potential risk of LVOTO.^7,18^

CT assessment is considered as the gold standard for risk stratification in TMVR patients.^19^ Recent studies, by demonstrating both an excellent correlation between the estimated neo-LVOT and actual neo-LVOT area with a pre-post procedural CT comparison and an inverse correlation between peak LVOT gradient and predicted neo-LVOT area, allowed the validation of this technique.^20,21^

In our study, 3D-TOE demonstrated the ability to identify ineligible patients for TMVR implantation with an almost perfect agreement between CT and 3D-TOE based on MA dimensions and LVOT measurements. This agreement is also maintained within the subgroups: native annulus, ViMAC, ViV, and ViR.

Despite our data showed a high correlation between the two methods, they also confirmed previous findings regarding the slight tendency of echocardiography to underestimate measurements compared to CT.^22–23^ In this regard, 3D-TOE, underestimating and predicting smaller neo-LVOT areas, may theoretically overestimate the risk of LVOTO, causing a possible incorrect exclusion of the patient for ineligibility. Similarly, it can lead to wrongly judge a MA too small for a TMVR. However, it appears reasonable, in order to not improperly exclude patients whose anatomical features are unsuitable for implantation by only a few mm in diameter or a few tenths of a cm^2^ in area, that a complementary CT evaluation is recommended to confirm the screening results.

In this regard, a possible approach could be to use the 95% limits of agreement of Bland-Altman test. In our study the 95% limits of agreement of Bland-Altman test between the CT and 3D-TOE lie always between less than 5 mm in MV diameters and less than 0.5 cm^2^ in neo-LVOT areas (*Table 2*). Based on these data, a patient with a difference between the 3D-TOE measured parameter and the limit set by manufacturer recommendations greater than the 95% limits of agreement may be judged unsuitable without CT (for example neo-LVOT measured by 3D-TOE 1.1 cm^2^, neo-LVOT manufacturer recommendations lower limit 1.7 cm^2^, difference 0.6 cm^2^ greater than 0.5 cm^2^). Conversely when the same difference is under the 95% limits of agreement a further screening assessment with CT-scan is mandatory to avoid possible incorrect exclusion in this gray zone (for example neo-LVOT measured by 3D-TOE 1.3 cm^2^, neo-LVOT manufacturer recommendations lower limit 1.7 cm^2^, difference 0.4 cm^2^ less than 0.5 cm^2^).

Bearing in mind these limits, our results showed that a comprehensive echocardiographic screening for TMVR with post-processing 3D-TOE dedicated software seems reliable as first screening methods to detect the patients with an anatomy unsuitable for TMVR, avoiding unnecessary CT evaluation. In our study cohort it is not surprising that 42% of patients presented chronic kidney disease and would have benefited of an alternative screening algorithm that did not need contrast medium utilization. Moreover, echocardiography being widely available, with lower costs and less side effects, on paper, represents the ideal modality for the screening of valvular interventions. On the other hand, it is operator dependent technique and relies on the proper acquisition and analysis of the images, so inevitably it presents an intrinsic higher likelihood of committing bias. However, as confirmed by our data and previous studies,^24,25^ the interobserver’s consistency and reproducibility of the data could dramatically increase through the rapid improvement of both 3D-TOE hardware and software allowing the increasing use of automated measurements.^26^

### Limitations

The results of the present analysis must be interpreted considering some limitations. The scenario of a real-world cohort brings the advantage of wide data generalizability but invariably is associated with inclusion bias. Poor image quality is still an issue for 3D-TOE analysis: excluding patients with low spatial resolution and low frame rate, permitted the analysis of only good quality images, introducing a possible selection bias. However, in our study all patients presented sufficient image quality and no one was excluded. Moreover, the presence of extensive mitral calcification was not a limitation for echocardiographic assessment in this subgroup of patients. A separate analysis of patients with calcifications in LVOT was not possible because they were not sufficiently represented in our population. Finally, the high reproducibility of these measurements is dependent on training and experience, thus our findings cannot necessarily be generalized to less-experienced readers.

## Conclusion

A comprehensive 3D-TOE screening for TMVR is feasible and reliable, showing good accuracy when compared to CT and might be proposed as the first-line imaging modality for TMVR screening to detect patients with certainly inappropriate anatomy. Due to the limited number of patients and the novel nature of the investigation, further studies are warranted to verify these preliminary findings.

## Supplementary data

Supplementary data are available at *European Heart Journal - Cardiovascular Imaging* online.

## Supplementary Material

jead015_Supplementary_Data

## Data Availability

The data underlying this article are available in the article and in its online Supplementary material.
