# Supplementary material for: Feasibility and reliability of comprehensive three-dimensional transoesophageal echocardiography screening process for transcatheter mitral valve replacement
Source: Eur Heart J Cardiovasc Imaging. 2023 Feb 17;24(8):1043–51. doi: 10.1093/ehjci/jead015 (PMC10364620; doi:10.1093/ehjci/jead015)
Supplement: jead015_Supplementary_Data [file jead015_supplementary_data.docx]

# SUPPLEMENTARY MATERIALS

## *
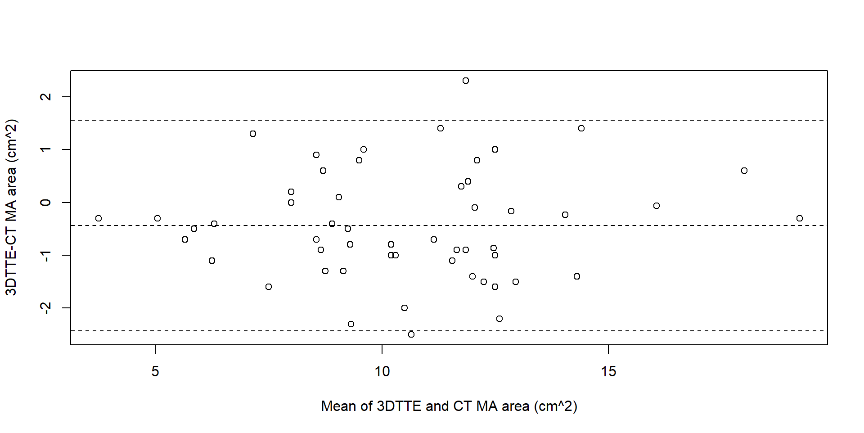
SUPPLEMENTARY GRAPHS*


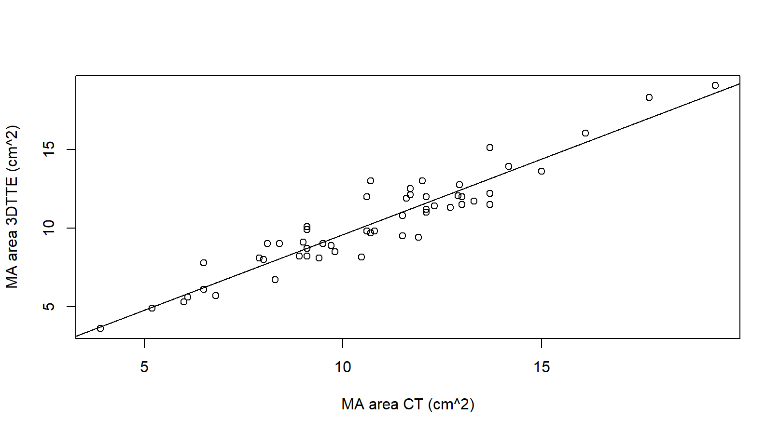


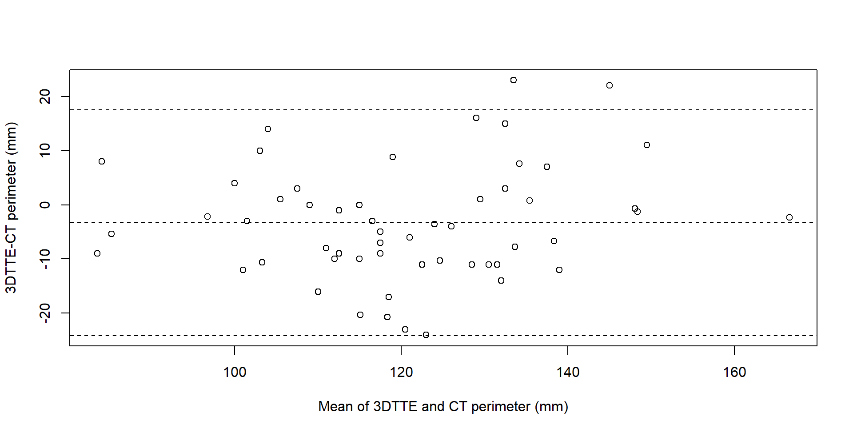

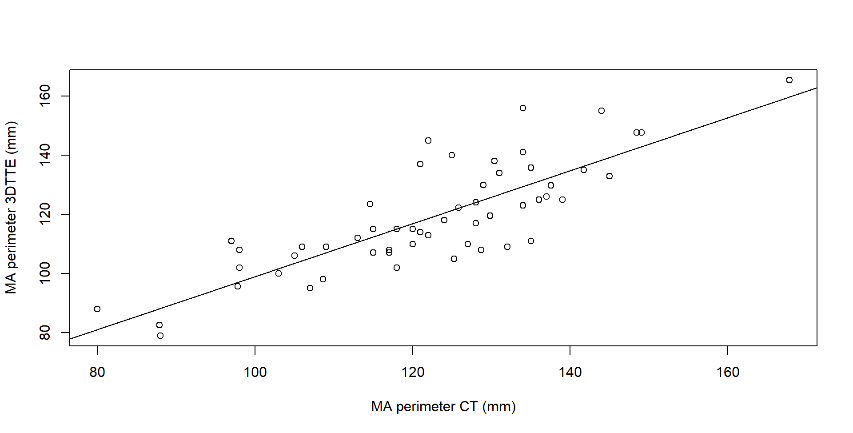

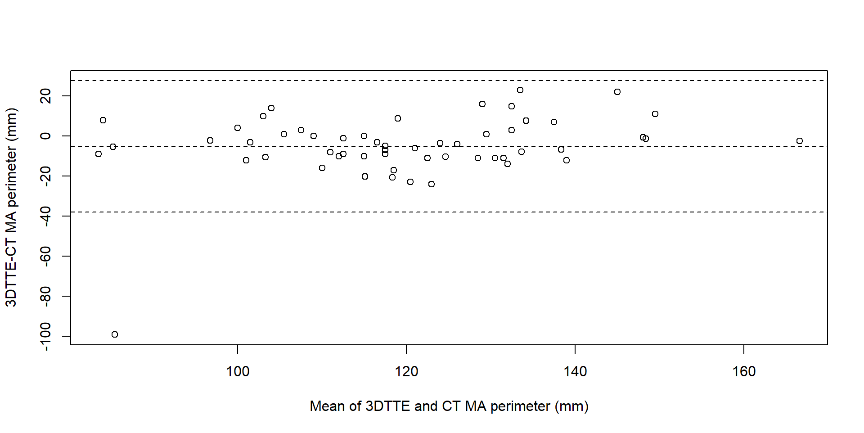
**Supplementary Graph 1**. Linear regression and Bland-Altman plot analysis between 3DTOE and CT for MA Area measurements


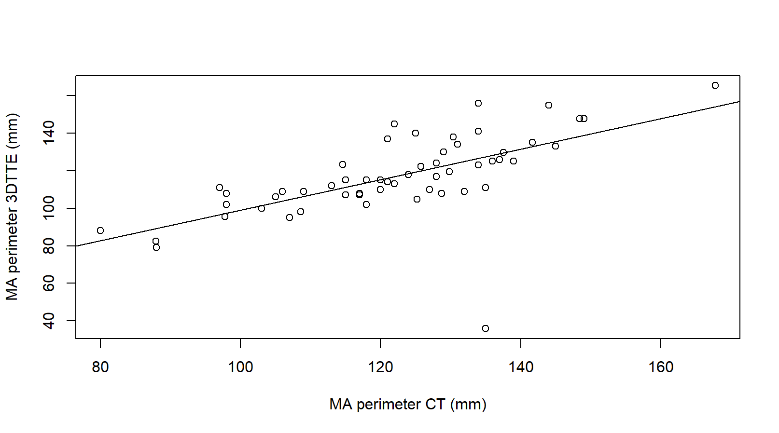


**Supplementary Graph 2.** Linear regression and Bland-Altman plot analysis between 3DTOE and CT for MA perimeter measurements


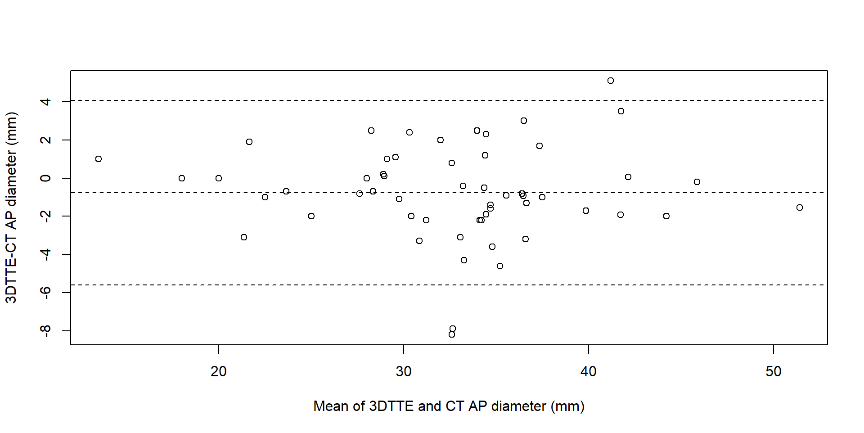

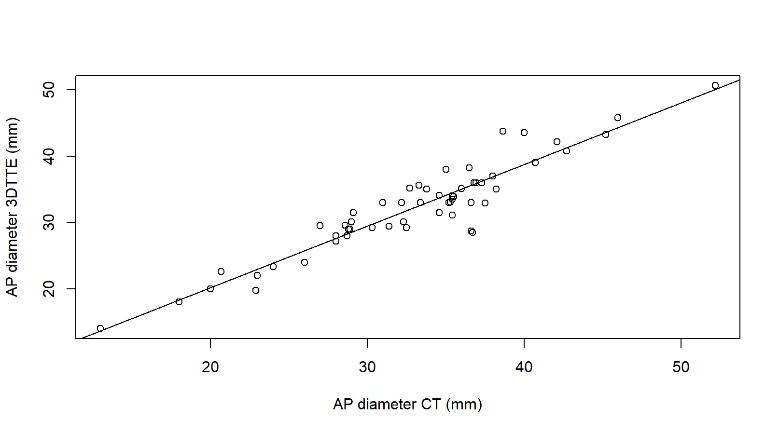


**Supplementary Graph 3.** Linear regression and Bland-Altman plot analysis between 3DTOE and CT for AP diameter measurements


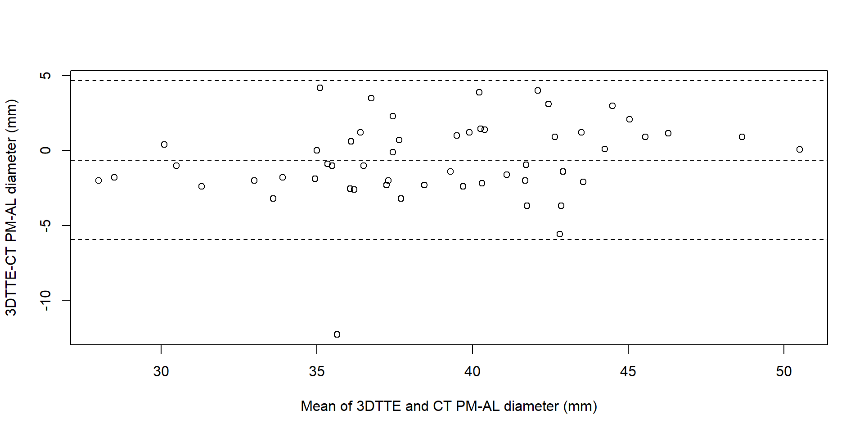

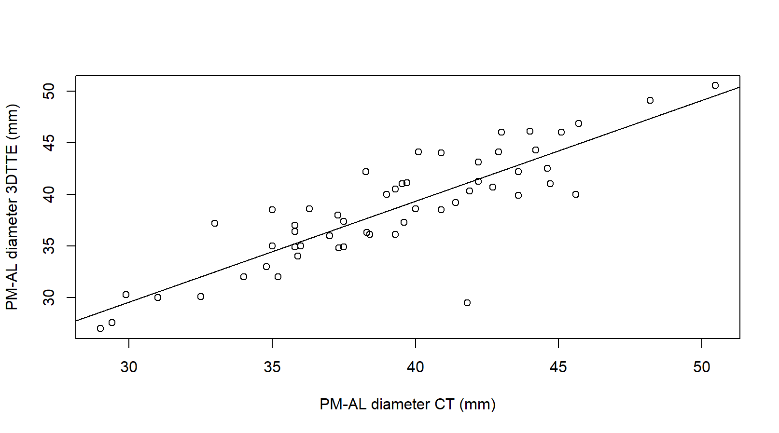
**Supplementary Graph 4.** Linear regression and Bland-Altman plot analysis between 3DTOE and CT for PM-AL diameter measurements


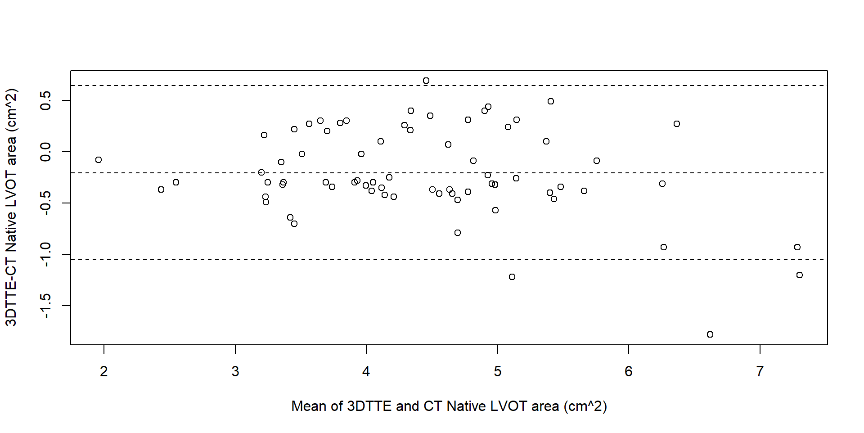


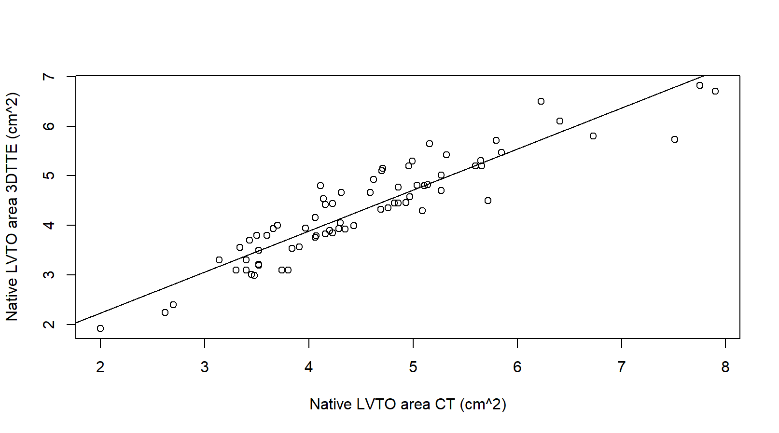
**Supplementary Graph 5.** Linear regression and Bland-Altman plot analysis between 3DTOE and CT for native LVOT area measurements.


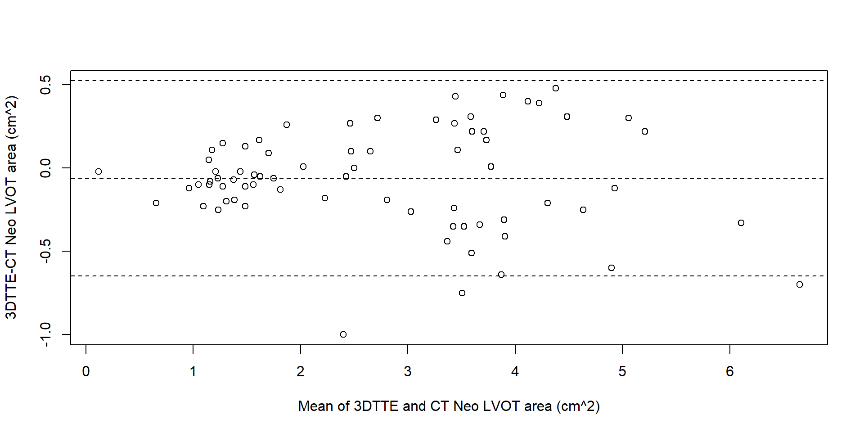

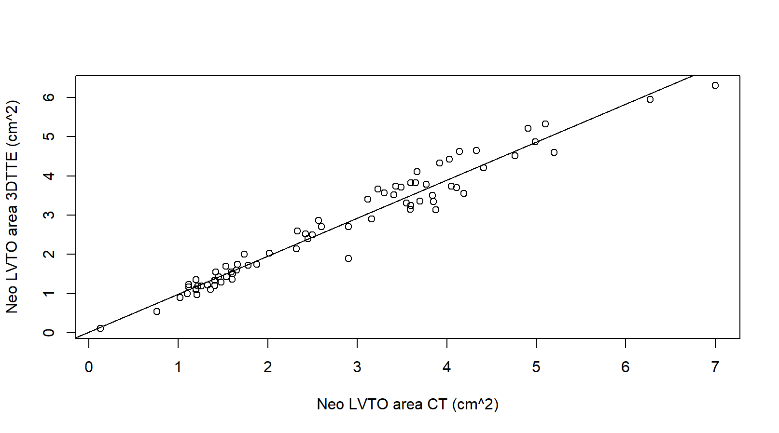


**Supplementary Graph 6.** Linear regression and Bland-Altman plot analysis between 3DTOE and CT for estimated Neo-LVOT area measurements.


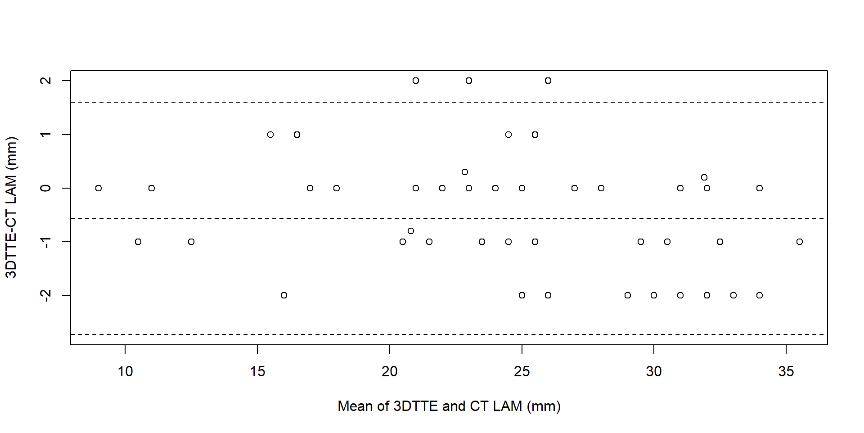

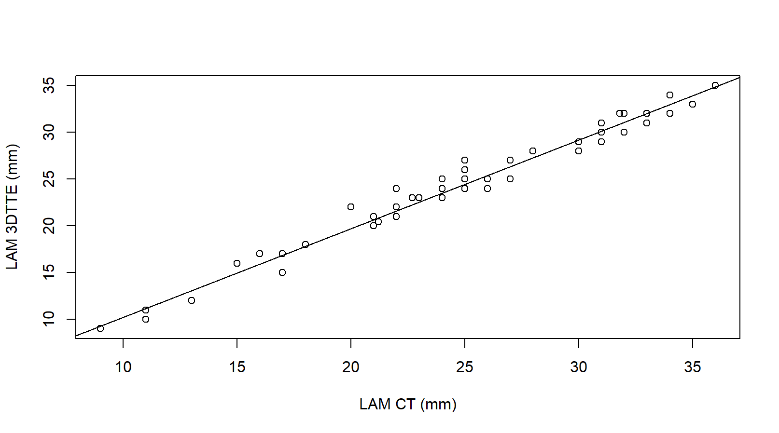
**Supplementary Graph 7.** Linear regression and Bland-Altman plot analysis between 3DTOE and CT for estimated AML length.

## SUPPLEMENTARY TABLES

### Supplementary Table 1: Correlation between 3D-TOE and CT measurements in subgroups

|  | **ViV and ViR 16pts** | | **ViMAC 16 pts** | | **TMVR in native annulus without MAC 40 pts** | |
| --- | --- | --- | --- | --- | --- | --- |
|  | **R** | **P** | **R** | **P** | **R** | **P** |
| Native LVOT area (cm^2^) | 0.88 | <0.0001 | 0.87 | <0.0001 | 0.82 | <0.0001 |
| Neo-LVOT area (cm^2^) | 0.90 | <0.0001 | 0.92 | <0.0001 | 0.97 | <0.0001 |
| Projected MA area (cm^2^) |  |  | 0.95 | <0.0001 | 0.80 | <0.0001 |
| Projected MA perimeter (mm) |  |  | 0.70 | <0.001 | 0.55 | <0.0001 |
| AP diameter (mm) |  |  | 0.91 | <0.0001 | 0.80 | <0.0001 |
| PM-AL diameter (mm) |  |  | 0.95 | <0.0001 | 0.71 | <0.0001 |
| AML lenght (mm) |  |  | 0.96 | <0.0001 | 0.97 | <0.0001 |

R is the Pearson’s correlation coefficient

Supplementary table 2: Accuracy of 3D-TOE in native anulus screening compared with CT as reference standard

|  | 3D-TOE screening Positive | 3D-TOE screening Negative | **Total** |
| --- | --- | --- | --- |
| CT screening positive | 24 | 3 | 27 |
| CT screening negative | 2 | 27 | 29 |
| **Total** | 26 | 30 | 56 |

|  | **3D-TOE** |
| --- | --- |
| Sensitivity (95% Cl) | 0.90 (0.73, 0.98) |
| Specificity (95% Cl) | 0.92 (0.75, 0.99) |
| Accuracy (95% Cl) | 0.91 (0.80, 0.97) |
| Cohen kappa (95% Cl) | 0.82 (0.67, 0.97) |
| Positive predictive value  (95% Cl) | 0.93 (0.77, 0.99) |
| Negative predictive value  (95% Cl) | 0.89 (0.71, 0.98) |
| Mcnemar's Test p-Value | 1 |

Supplementary Table 3: Accuracy of 3D-TOE in VIV and VIR screening compared with CT as reference standard

|  | 3D-TOE screening Positive | 3D-TOE screening Negative | **Total** |
| --- | --- | --- | --- |
| CT screening positive | 13 | 0 | 13 |
| CT screening negative | 1 | 2 | 3 |
| **Total** | 14 | 2 | 16 |

|  | **3D-TOE** |
| --- | --- |
| Sensitivity (95% Cl) | 1.00 (0.16, 1.00) |
| Specificity (95% Cl) | 0.93 (0.66, 1.00) |
| Accuracy (95% Cl) | 0.94 (0.70, 0.99) |
| Cohen kappa (95% Cl) | 0.76 (0.32, 1.21) |
| Positive predictive value  (95% Cl) | 0.67 (0.09, 0.99) |
| Negative predictive value  (95% Cl) | 1.00 (0.75, 1.00) |
| Mcnemar's Test p-Value | 1 |

Supplementary Table 4: Accuracy of 3D-TOE in ViMAC screening compared with CT as reference standard

|  | 3D-TOE screening Positive | 3D-TOE screening Negative | **Total** |
| --- | --- | --- | --- |
| CT screening positive | 3 | 1 | 4 |
| CT screening negative | 0 | 12 | 12 |
| **Total** | 3 | 13 | 16 |

|  | **3D-TOE** |
| --- | --- |
| Sensitivity (95% Cl) | 0.92 (0.64, 1.00) |
| Specificity (95% Cl) | 1.00 (0.29, 1.00) |
| Accuracy (95% Cl) | 0.93 (0.70, 0.99) |
| Cohen kappa (95% Cl) | 0.82 (0.47, 1.16) |
| Positive predictive value  (95% Cl) | 1.00 (0.74, 1.00) |
| Negative predictive value  (95% Cl) | 0.75 (0.19, 0.99) |
| Mcnemar's Test p-Value | 1 |

**Supplementary Table 5:** Accuracy of 3D-TOE in Neo-LVOT area assessment compared with CT as reference standard

|  | 3D-TOE NeoLVOT >1.7 cm2 | 3D-TOE NeoLVOT <1.7 cm2 | Total |
| --- | --- | --- | --- |
| CT neoLVOT >1.7 cm2 | 46 | 0 | 46 |
| CT neoLVOT <1.7 cm2 | 2 | 24 | 26 |
| Total | 48 | 24 | 72 |

|  | 3D-TOE |
| --- | --- |
| Sensitivity (95% Cl) | 1.00 (0.86, 1.00) |
| Specificity (95% Cl) | 0.96 (0.86, 0.99) |
| Accuracy (95% Cl) | 0.97 (0.90, 0.99) |
| Cohen kappa (95% Cl) | 0.94 (0.86- 1.02) |
| Positive predictive value (95% Cl) | 0.92 (0.75, 0.99) |
| Negative predictive value (95% Cl) | 1.00 (0.92, 1.00) |
| Mcnemar's Test p-Value | 0.48 |

**Supplementary Table 6:** Accuracy of 3D-TOE in too small mitral anulus assessment compared with CT as reference standard in native TMVR.

|  | 3D-TOE not too small MA | 3D-TOE too small MA | Total |
| --- | --- | --- | --- |
| CT not too small MA | 41 | 6 | 47 |
| CT too small MA | 1 | 6 | 7 |
| Total | 42 | 12 | 54 |

|  | 3D-TOE |
| --- | --- |
| Sensitivity (95% Cl) | 0.50 (0.21, 0.79) |
| Specificity (95% Cl) | 0.98 (0.87, 1.00) |
| Accuracy (95% Cl) | 0.87 (0.75- 0.94) |
| Cohen kappa (95% Cl) | 0.56 (0.25- 0.86) |
| Positive predictive value(95% Cl) | 0.86 (0.42, 1.00) |
| Negative predictive value (95% Cl) | 0.87 (0.74, 0.95) |
| Mcnemar's Test p-Value | 0.13 |

**Supplementary Table 7:** Accuracy of 3D-TOE in too large mitral anulus assessment compared with CT as reference standard in native TMVR.

|  | 3D-TOE not too large MA | 3D-TOE too large MA | Total |
| --- | --- | --- | --- |
| CT not too large MA | 52 | 0 | 52 |
| CT too large MA | 0 | 2 | 2 |
| Total | 52 | 2 | 54 |

|  | 3D-TOE |
| --- | --- |
| Sensitivity (95% Cl) | 1.00 (0.16, 1.00) |
| Specificity (95% Cl) | 1.00 (0.93, 1.00) |
| Accuracy (95% Cl) | 1.00 (0.93- 1.00) |
| Cohen kappa (95% Cl) | 1 |
| Positive predictive value (95% Cl) | 1.00 (0.16, 1.00) |
| Negative predictive value (95% Cl) | 1.00 (0.93, 1.00) |
| Mcnemar's Test p-Value | Na |

**Supplementary Table 8:** Screening difference among anatomical CT criteria screening and manufacturer screening based on anatomical CT criteria and clinical conditions

| Total population | Approved by anatomical CT criteria | Approved by manufacturer | Reasons for this difference | |
| --- | --- | --- | --- | --- |
| 72 (16 VIV and VIR, 56 TMVR in native anulus) | 40 | 44 | Manufacturer did not approve:  -2 pts for comorbidities  -3 pts with low fossa ovalis | Manufacturer approved:  -2 pts with small MA according to CT anatomical criteria  -3 pts with big MA according to CT anatomical criteria  - 4 pts with small neo-LVOT according to CT anatomical criteria |

|  | Inter-observer | | Intra-observer | |
| --- | --- | --- | --- | --- |
| Variable | ICC | P value | ICC | P value |
| Annulus Area | 0.974 | <0.0001 | 0.984 | <0.0001 |
| Perimeter | 0.966 | <0.0001 | 0.980 | <0.0001 |
| AP diameter | 0.942 | <0.0001 | 0.953 | <0.0001 |
| PM-AL diameter | 0.972 | <0.0001 | 0.960 | <0.0001 |
| LVOT Area | 0.873 | <0.0001 | 0.942 | <0.0001 |
| NeoLVOT | 0.896 | <0.0001 | 0.918 | <0.0001 |

**Supplementary Table 5:** Interobserver and intraobserver variability for 3DTOE measurements:
